# Damage Control Management for Thoracic Trauma with Cardiac Arrest Complicated by Emphysematous Gastritis and Cystitis

**DOI:** 10.7759/cureus.7102

**Published:** 2020-02-25

**Authors:** Shunsuke Madokoro, Youichi Yanagawa, Hiroki Nagasawa, Ikuto Takeuchi, Yasumasa Oode

**Affiliations:** 1 Acute Critical Care Medicine, Juntendo University Shizuoka Hospital, Izunokuni, JPN; 2 Intensive and Emergency Medicine, Juntendo University Shizuoka Hospital, Izunokuni, JPN

**Keywords:** damage control surgery, thoracic trauma, emphysematous gastritis, emphysematous cystitis

## Abstract

A 78-year-old man was found unconscious after sliding from a rock. His history included hypertension, atrial fibrillation and cerebral infarction requiring warfarin. On arrival, he received six units of blood type O transfusion and vitamin K in an emergency room (ER) due to hemorrhagic shock. His systolic blood pressure temporarily increased to 100 mmHg, and he underwent traumatic pan scan revealing occipital fracture, cerebral contusion, and cervical and multiple left rib fractures with left-dominant bilateral hemothorax. He re-entered a shock state after the examination and underwent transfusion again, but he then entered cardiac arrest. He underwent damage control surgery in the ER and obtained spontaneous circulation. The postoperative course was eventful, but he eventually obtained a survival outcome. Damage control surgery may be beneficial, even in cases of severe thoracic blunt trauma; however, postoperative infections may cause severe problems.

## Introduction

Damage control surgery (DCS) is an integral part of the management of critically injured patients. It usually involves patients with profound hemorrhagic shock who have developed acidosis, hypothermia and coagulopathy. Three stages of DCS are widely accepted: (1) limited operation to control bleeding and contamination, (2) continued resuscitation in the intensive care unit and (3) reoperation. In addition to cases of intraabdominal trauma, DCS techniques are also employed for managing thoracic injuries [1]. However, damage control techniques in thoracic trauma have been infrequently reported [2,3].

We herein report a case of damage control management for a patient with transient cardiac arrest due to severe thoracic trauma in the emergency department, complicated by an eventful postoperative course, including emphysematous gastritis and cystitis infection, but with a survival outcome.

## Case presentation

A 78-year-old man slipped and fell into a swamp and was found to be unconscious after hitting his head on a rock during the daytime in spring. His history included hypertension, atrial fibrillation and cerebral infarction requiring warfarin. A physician-staffed helicopter was dispatched at the request of the fire department. When the staff of the helicopter checked him at the rendezvous point, he was in a deep coma and shock state with subcutaneous emphysema at his left chest, so a venous route was secured, and tracheal intubation and left thoracostomy were performed before air evacuation. Left thoracostomy was made by the insertion of a chest drainage tube at the fourth intercostal space on the midaxillary line due to potential tension pneumothorax. During air transportation, he remained in a shock state due to minimum infusion based on permissive hypotension strategy, and the physician of the helicopter activated the massive transfusion protocol at the receiving emergency room [4].

On arrival, his vital signs were as follows: Glasgow Coma Scale, E1VTM1 without sedation; blood pressure, 58/44 mmHg; heart rate, 120 beats per minute; percutaneous saturation, 90% (FiO_2_ 1.0); body temperature, 33.7°C. The results of a venous gas analysis on arrival were as follows: pH, 7.353; PCO_2_, 41.7 mmHg; PO_2_, 38.9 mmHg; HCO_3_^-^, 22.6 mmol/L; base excess. -2.3 mmol/L; and lactate, 3.8 mmol/L. After receiving six units of blood type O transfusion and vitamin K, his systolic blood pressure temporarily increased to 100 mmHg, and he underwent traumatic pan scan.

**Figure 1:**
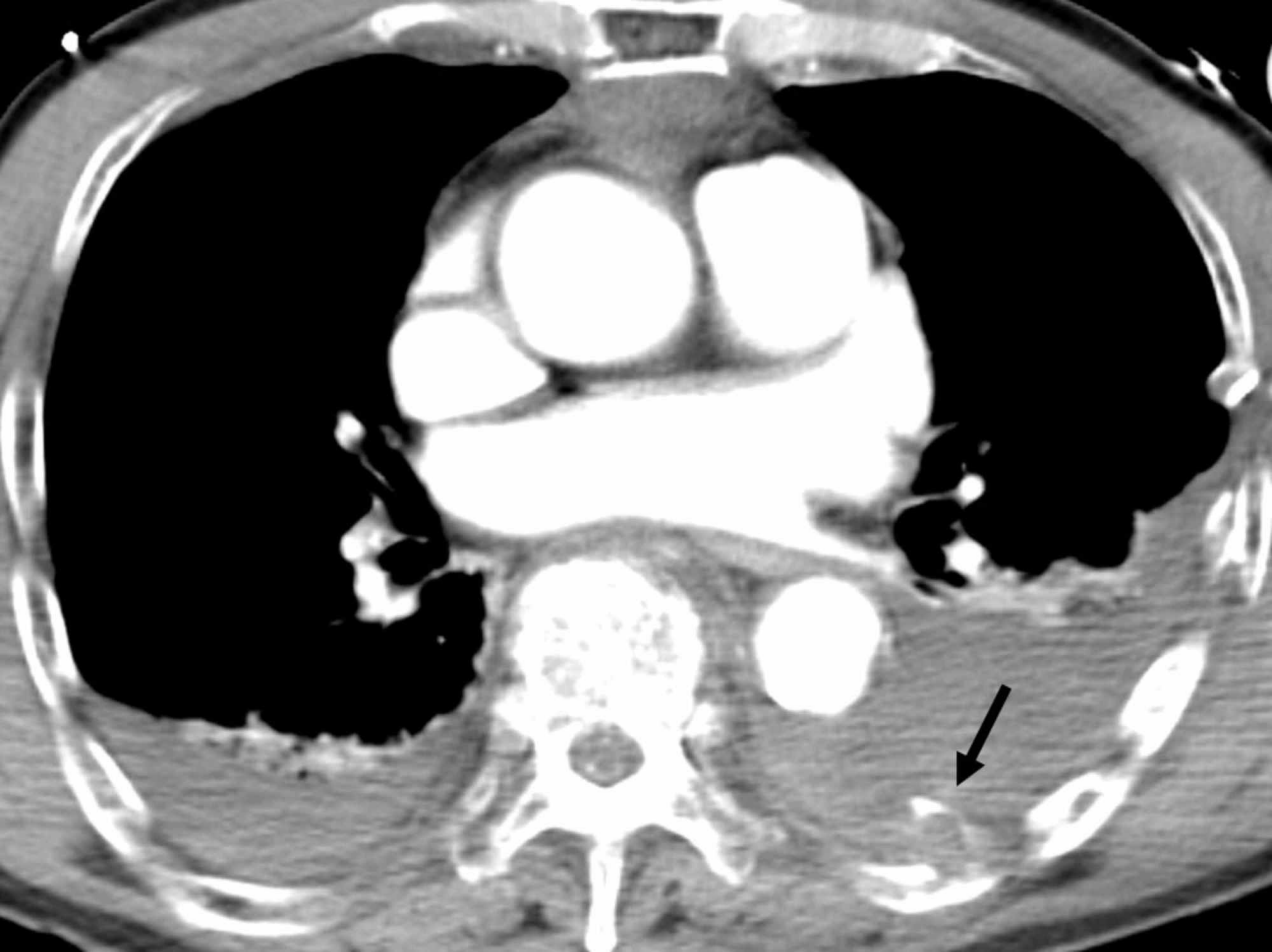
Computed tomography (CT) on arrival CT revealed multiple left rib and sternal fractures with left-dominant bilateral hemothorax and anterior mediastinal hematoma. The figure shows extravasation of contrast medium (arrow).

Computed tomography (CT) revealed occipital fracture, cerebral contusion, cervical fracture (C2-C4), multiple left rib and sternal fractures with left-dominant bilateral hemothorax (Figure 1).

He entered a shock state again after the CT examination and underwent transfusion again, but he then entered cardiac arrest. He underwent emergency bilateral thoracotomy with a normal surgical knife (no electrocautery) at the spine position. The initial bleeding amount in the bilateral thorax exceeded 1 L in addition to over 500 mL of blood that was drained via the chest drainage tube before thoracotomy. A bleeding tendency was also recognized at the skin incision sites. Gauze was packed into multiple bleeding sites, including venous hemorrhaging induced by the rib fractures, and the chest was closed. During damage surgery, he obtained spontaneous circulation after massive transfusion and the administration of fresh-frozen plasma, cryoprecipitate and human prothrombin complex.

The results of a biochemical blood analysis on arrival were as follows: white blood cell count, 15,000/μL; hemoglobin, 13.0 g/dL; platelet count, 6.0×10^4^/μL; total protein, 4.1 g/dL; glucose, 357 mg/dL; HbA_1_C, 6.3%; total bilirubin, 0.7 mg/dL; aspartate aminotransferase, 38 IU/L; alanine aminotransferase, 20 IU/L; blood urea nitrogen, 23.1 mg/dL; creatinine, 1.13 mg/dL; sodium, 140 mEq/L; potassium, 4.8 mEq/L; chloride, 106 mEq/L; C-reactive protein, 0.04 mg/dL; prothrombin time international normalized ratio, 2.91; activated partial thromboplastin time, 85.0 (27.0) seconds; fibrinogen, 80 mg/dL; and fibrinogen fibrin degradation product, 960 μg/mL. He was transferred to the intensive care unit.

He received 36 units of packed red blood cells, 16 units of fresh-frozen plasma and 30 units of packed platelets to maintain circulation after admission. On the second hospital day, he received a second-look operation for the removal of the gauze, hemostasis, irrigation of thoracic cavity and placement of chest drainage tubes in the operation room. On the sixth hospital day, he became complicated with pneumonia and underwent tracheostomy. He also was complicated with thrombotic microangiopathy, resulting in multiple cerebral infarctions and foot necrosis. After these complications had improved, he regained consciousness and achieved spontaneous breathing without mechanical ventilation and stable circulation, resulting in his being able to walk with assistance, at least for a little while.

**Figure 2:**
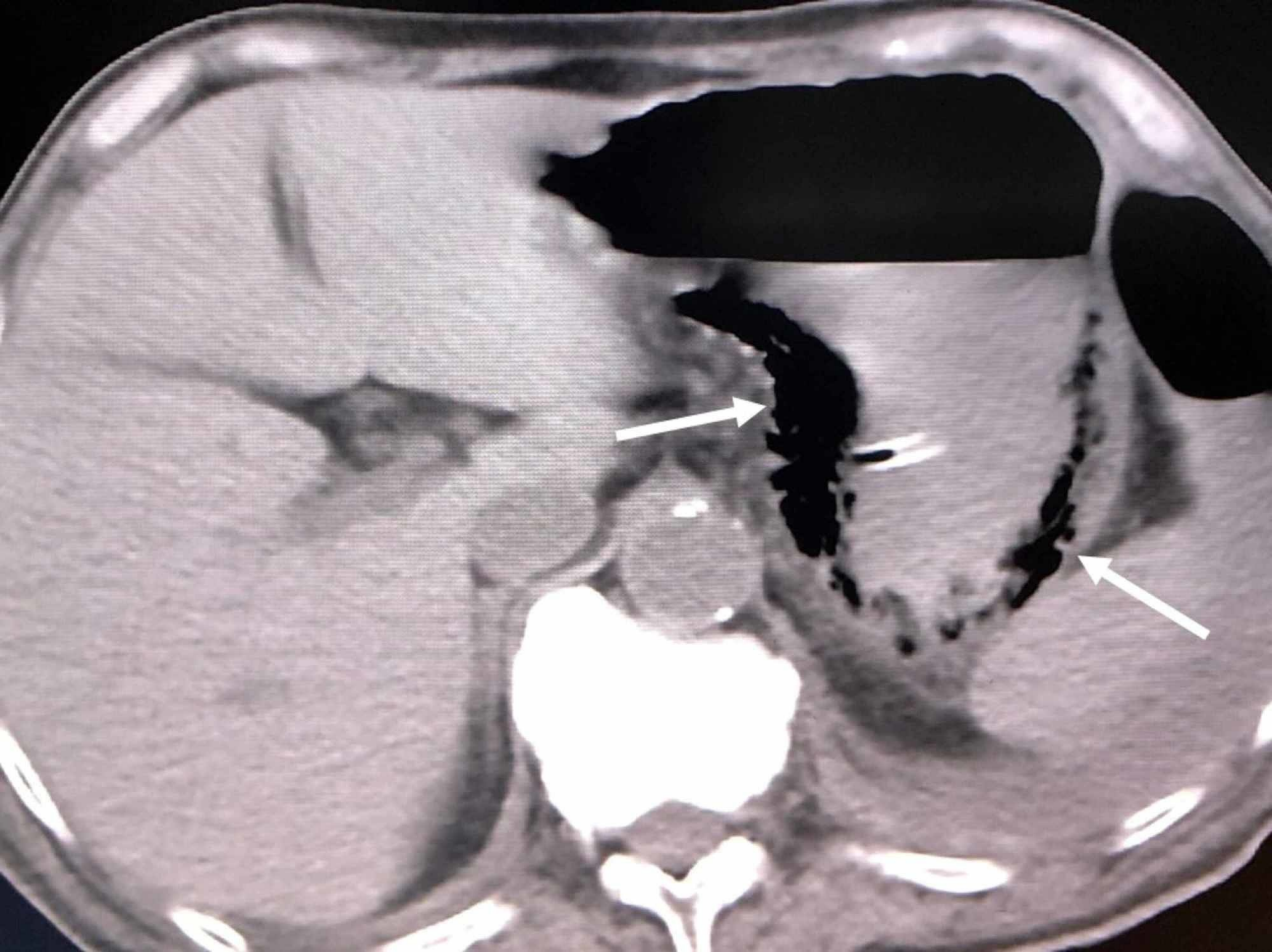
Computed tomography (CT) on the 28th hospital day (arrows) CT revealed left pyothorax and emphysematous gastritis.

However, he became septic and developed hypoxia again, and CT and gastroscopy on the 28th hospital day revealed left pyothorax and emphysematous gastritis (Figure 2). Cultures of both pyothorax and blood showed extended-spectrum β-lactamase (ESBL) Escherichia coli, which was treated by drainage and carbapenem administration.

**Figure 3:**
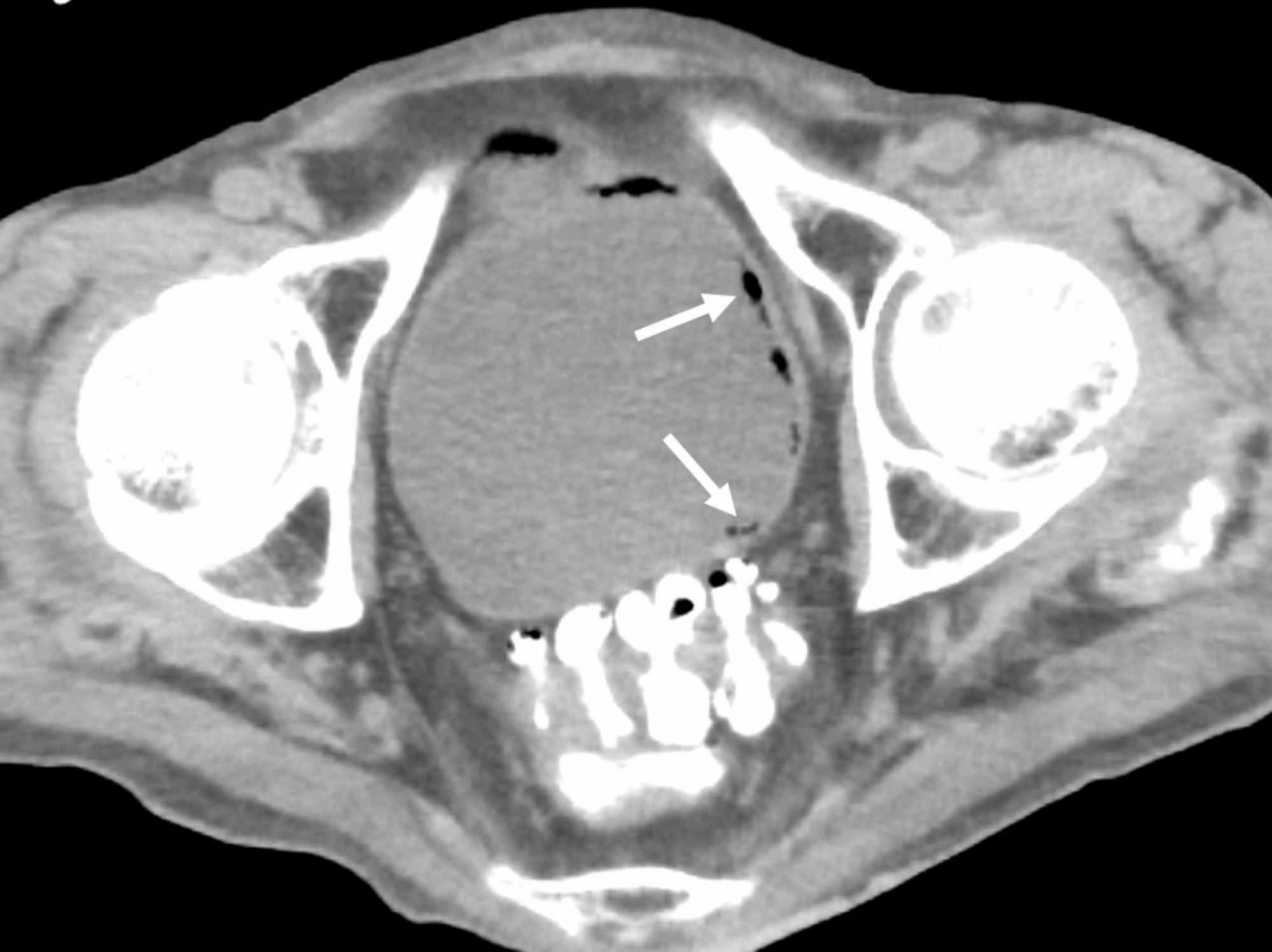
Computed tomography (CT) on the 81st hospital day CT revealed emphysematous cystitis (arrows).

Pyothorax and emphysematous gastritis improved, but he became complicated with repeated aspiration pneumonia after starting oral intake. CT on the 81st hospital day for evaluation of a recurrent fever revealed emphysematous cystitis (Figure 3), which was treated by indwelling balloon and carbapenem administration again. The culture of the urine revealed ESBL E. coli.

After these treatments, his vital signs stabilized. He was transferred to another hospital for rehabilitation of dysphagia and disused muscle atrophy.

## Discussion

This is the unique case of damage control management for a patient with transient cardiac arrest in the emergency department in which a survival outcome was obtained despite complication with an eventful postoperative course. Respiratory surgeons previously introduced damage control procedures for chest injuries, including aortic cross-clamping, hilar clamping, major vessel ligation, pulmonary tractotomy, simultaneously stapled pneumonectomy or lobectomy, cardiac stapling, balloon catheter tamponade, temporary intraluminal shunt, towel packing, towel clip closure and/or single en masse closure of the chest wall [5]. However, the present case was initially managed by emergency physicians, so simple thoracotomy and gauze packing were performed.

As the patient had taken warfarin in advance, he already had the deadly triad of hypothermia, coagulopathy and metabolic acidosis at arrival. In addition, he became complicated with profound hemorrhagic shock, resulting in cardiac arrest, so we selected DCS for severe thoracic injuries in the emergency room. There might have been some criticism that the thoracic CT image in the present case showed only one bleeding point, and thus it was deemed to be easy to carry out hemostasis. However, multiple bleeding sites were actually observed after thoracotomy, even with the field of vision being limited due to the supine position, in addition to active bleeding from the skin incision sites.

The main issue with the present case was the development of postoperative infections, primarily pneumonia resulting in pyothorax, followed by emphysematous gastritis and cystitis. Resuscitative emergency room thoracotomy may have carried a risk of exposure and lethal infection; however, the actual rate of complication with infection after resuscitative emergency room thoracotomy is low, contrary to the present case [6-8]. Accordingly, emergency physicians should attempt to perform DCS when possible for severe thoracic injuries.

This case had two sites of infection with emphysematous gastritis and cystitis at different timings. This is the first report of such a combination of infections. The initial treatment using carbapenem for ESBL E. coli might have been insufficient in this patient; as a result, delayed emphysematous cystitis induced by ESBL E. coli might occur in the present case. However, CT was a useful modality for detecting such lethal complications and helped us decide how to change the treatment protocol.

## Conclusions

We reported the unique case of damage control management for a patient with transient cardiac arrest in the emergency department who obtained survival outcome despite complications and an eventful postoperative course. DCS may be beneficial even in cases of severe thoracic blunt trauma.
